# Extracellular Glutathione Peroxidase GPx3 and Its Role in Cancer

**DOI:** 10.3390/cancers12082197

**Published:** 2020-08-06

**Authors:** Caroline Chang, Beth L. Worley, Rébécca Phaëton, Nadine Hempel

**Affiliations:** 1 Department of Comparative Medicine, Penn State University College of Medicine, Hershey, PA 17033, USA; cchang1@pennstatehealth.psu.edu; 2 Department of Pharmacology, Penn State University College of Medicine, Hershey, PA 17033, USA; bworley@pennstatehealth.psu.edu; 3 Department of Obstetrics & Gynecology & Department of Microbiology and Immunology, Penn State University College of Medicine, Hershey, PA 17033, USA; rphaeton@pennstatehealth.psu.edu

**Keywords:** antioxidant enzymes, extracellular glutathione peroxidase, GPx3, GPx-P, eGPx, selenoproteins, glutathione, cancer, redox signaling, oxidative stress, reactive oxygen species

## Abstract

Mammalian cells possess a multifaceted antioxidant enzyme system, which includes superoxide dismutases, catalase, the peroxiredoxin/thioredoxin and the glutathione peroxidase systems. The dichotomous role of reactive oxygen species and antioxidant enzymes in tumorigenesis and cancer progression complicates the use of small molecule antioxidants, pro-oxidants, and targeting of antioxidant enzymes as therapeutic approaches for cancer treatment. It also highlights the need for additional studies to investigate the role and regulation of these antioxidant enzymes in cancer. The focus of this review is on glutathione peroxidase 3 (GPx3), a selenoprotein, and the only extracellular GPx of a family of oxidoreductases that catalyze the detoxification of hydro- and soluble lipid hydroperoxides by reduced glutathione. In addition to summarizing the biochemical function, regulation, and disease associations of GPx3, we specifically discuss the role and regulation of systemic and tumor cell expressed GPx3 in cancer. From this it is evident that GPx3 has a dichotomous role in different tumor types, acting as both a tumor suppressor and pro-survival protein. Further studies are needed to examine how loss or gain of GPx3 specifically affects oxidant scavenging and redox signaling in the extracellular tumor microenvironment, and how GPx3 might be targeted for therapeutic intervention.

## 1. Introduction

The cell is equipped with a large arsenal of antioxidants, including enzymes that have distinct affinities and specificities for exogenously and endogenously produced oxidants [1,2,3,4,5]. These include the superoxide (O_2_**^•^**^−^) dismutases (SOD), catalase (CAT) responsible for hydrogen peroxide (H_2_O_2_) scavenging, and the glutathione peroxidase (GPx) and peroxiredoxin (PRDX)/thioredoxin (TXN) systems, with substrate affinity for hydroperoxides [2,3]. Besides catalase, each enzyme family is comprised of multiple members that display distinct cellular localization, including organellar compartmentalization that helps position them close to the source of oxidant production [1,2,3]. An example is SOD2, which is localized within the mitochondrial matrix and is primarily responsible for the scavenging of O_2_^•−^ produced at complex I and III of the electron transport chain. While the majority of antioxidant enzymes are localized within intracellular organelles and the cytosol, copper zinc superoxide dismutase (SOD3/CuZnSOD; for reviews see [6,7]) and glutathione peroxidase 3 (GPx3) represent two enzymes with extracellular localization [5].

It is now widely appreciated that antioxidant enzymes play an important dichotomous role in cancer. Many antioxidant enzymes protect cells from tumor initiation and the process of carcinogenesis. This is largely due to their role in preventing the accumulation of deleterious levels of oxidants that elicit macromolecular damage, including DNA oxidation that results in mutations and tumor initiation. Hence, many antioxidant enzymes were originally classified as tumor suppressors [6]. Conversely, it has been demonstrated that many cancer cells require oxidant scavenging and the upregulation of antioxidant enzyme expression for tumor progression and metastasis [8,9,10,11,12,13,14]. These dichotomous roles of antioxidants at different tumor stages highlight the need for additional studies investigating the role, interplay and regulation of antioxidant enzymes in cancer. Moreover, it is important to consider the antioxidant landscape of tumor cells for the development of therapeutic strategies, which accordingly may involve either antioxidant or pro-oxidant approaches, or the targeting of antioxidant enzyme expression (reviewed in [15,16,17]).

This review focuses on GPx3, which has received relatively little attention in the cancer field. We highlight studies focused on the role and regulation of GPx3 in cancer, at both the systemic level and in response to altered expression by tumor cells.

## 2. Extracellular Glutathione Peroxidase GPx3

### 2.1. GPx3 Function

**Figure 1:**
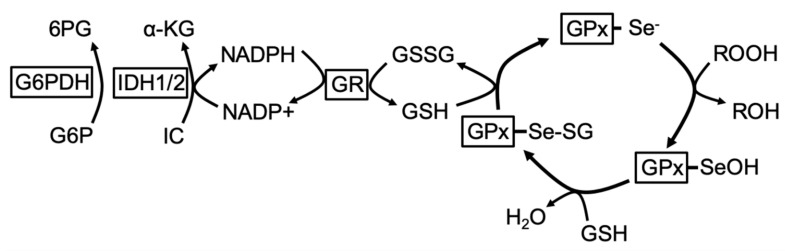
Schematic of the reduction of hydroperoxides by selenocysteine-containing glutathione peroxidases (GPx). The selenolate state (GPx-Se-) reduces hydroperoxides to water to form the oxidized selenenic acid (GPx-SeOH). GPx-SeOH reacts with GSH to generate selenenyl sulfide (GPx-SeSG), which is regenerated to the active selenol following nucleophilic attack of a second GSH and formation of oxidized glutathione (GSSG). GSSG is reduced back to GSH by glutathione reductase (GR). Nicotinamide adenine dinucleotide phosphate (NADPH) reducing equivalents for this reaction are mainly derived from isocitrate dehydrogenases (IDH)1/2, including the forward reaction of IDH2 in the tricarboxylic acid (TCA) cycle, and via the reduction of glucose-6-phosphate (G6P) in the pentose phosphate pathway by glucose-6-phosphate dehydrogenase (G6PDH). (Enzymes are indicated by boxes; α-KG: α-ketoglutarate; IC: isocitrate; 6PG: 6-phospho-gluconate).

The glutathione peroxidase family consists of eight isozymes (GPx1–8) that catalyze the reduction of hydrogen peroxide, hydroperoxides, and lipid hydroperoxides by reduced glutathione (GSH). Of the eight glutathione peroxidases, five are selenocysteine-containing proteins (GPx1–4, GPx6) [18]. The human genome encodes 25 selenocysteine-containing proteins, or selenoproteins, that contain the amino acid selenocysteine (Sec) in their polypeptide chain. Sec is the only amino acid with an essential dietary micronutrient as a constitutive component. Sec is encoded by the UGA codon, which normally signals the end of translation, and its translation occurs via its own tRNA [19,20,21,22]. Many selenoproteins are essential for embryonic development, yet only 13 have known functions [23]. Of these, glutathione peroxidases, thioredoxin reductases (TXNRD), and thyroid hormone deiodinases (DIO) are known to be involved in redox regulation of intracellular signaling, redox homeostasis, and thyroid hormone metabolism [19]. The human *GPX3* gene is made up of five exons spanning 10 kb in the 5q32 region of chromosome 5 [24,25], encoding a 23 kDA protein that forms a homotetramer [24]. Each monomer contains a selenocysteine at the enzyme active site, which is also composed of a glutamine, tryptophan, and asparagine to constitute a conserved tetrad [26]. In GPxs, hydroperoxides such as H_2_O_2_ oxidize the selenol (–SeH) group of Sec to selenenic acid (–SeOH), which is then reduced by glutathione. The proposed mechanism for the reduction of hydroperoxides by seleno-containing GPx enzymes is illustrated in Figure 1, which is based on functional studies of GPx1 activity [27]. GPx3 catalyzes the reduction of hydroperoxides, including H_2_O_2_ and soluble lipid hydroperoxides, by reduced glutathione [28,29,30]. Enzyme kinetics of GPx3 towards H_2_O_2_ are similar to those of cellular GPx1 (k1 of human plasma isolated GPx3, 4 × 10^7^ M^−1^sec^−1^) [30]. Unlike GPx1, GPx3 can also utilize soluble lipid hydroperoxides as substrates, similar to GPx4 [30]. However, the specific substrates relevant to GPx3′s role in normal physiological function and during disease progression need to be identified and GPx3 enzyme kinetics further explored.

GSH is synthesized in the cell cytosol and exported into the plasma as a major pathway of GSH degradation [31]. Since GSH is rapidly catabolized while in circulation, the available plasma concentration of GSH (~10 μM) is relatively low compared to intracellular GSH levels (~1–10 mM) [31]. This has the potential to significantly limit GPx3 activity. Thus, the role of GPx3 as a major systemic antioxidant enzyme has been questioned. Instead, it has been proposed that GPx3, similar to selenoprotein-P, may act at hubs of reactive oxygen species (ROS) generation, including enhanced lipid hydroperoxide generation within the vicinity of lipoxygenases (LOX) at the cell surface [32,33]. However, this requires further experimental validation. These localized hubs of GPx3 activity would also require a local supply of GSH. Indeed, it is known that specific sites, such as the hepatic vein plasma and the epithelial lining fluid of the lower respiratory tract, contain much higher concentrations of GSH compared to average plasma levels [34]. In addition, it has been demonstrated that cells secrete GSH in a localized manner in areas where GPx3 binds to the basement membrane [34,35]. Since GSH levels often increase in response to oxidative stress, this might represent a further regulatory mechanism of GPx3 activity [36]. Alternatively, it has been suggested that in situations of GSH depletion, GPx3 is able to interact with and use glutaredoxin and thioredoxin reductase by itself or with thioredoxin, as alternate electron donors to GSH [37]. These situations may create microenvironments that favor enhanced GPx3 activity.

To gain functional insights into the role of GPx3, *GPX3* global knock-out mice have been generated in a C57BL/6 background [38]. Interestingly, while knock-out of other intracellular antioxidant enzymes, such as the mitochondrial superoxide dismutase SOD2, is embryonically lethal, homozygous loss of the extracellular antioxidant enzymes SOD3 and GPx3 does not affect viability [38,39,40]. The *GPX3-/-* knock-out mice are outwardly indistinguishable from wild-type mice when unchallenged. However, a noticeable phenotype of *GPX3-/-* knock-out mice that recapitulates observations in patients with low systemic GPx3 levels is the susceptibility to thrombosis following platelet activation. *GPX3-/-* mice display decreased bleeding time compared to wild-type mice, and this was mechanistically linked to enhanced oxidative inactivation of nitric oxide (NO) and consequential increases in thrombosis [41,42]. Moreover, when *GPX3-/-* knock-out mice were exposed to surgery-induced chronic kidney disease (CKD), these mice displayed decreased left ventricular fractional shortening, increased platelet aggregation, and microthrombi in the myocardium [41]. The pathogenesis leading from CKD to cardiovascular disease in *GPX3-/-* mice is thought to be from the significant oxidative stress promoted by CKD leading to endothelial dysfunction, decreased NO, and consequential vascular disease and thrombosis [41]. Similar to *GPX3-/-* knock-out mice, GPx3 overexpressing transgenic mice are outwardly normal. These mice have a 50% increase in plasma GPx3 activity, which was demonstrated to confer protection against systemic acetaminophen toxicity [43]. Interestingly, elevated GPx3 expression also made mice more thermosensitive, which was hypothesized to be due to a decrease in H_2_O_2_ and lipid hydroperoxides, both of which are inducers of heat shock protein 70 (HSP70), which protects against the cytotoxic effects of hyperthermia [44].

### 2.2. GPx3 Regulation

An important regulatory mechanism of selenocysteine-containing proteins is the availability of selenium (Se) [45,46]. Selenoproteins display a hierarchy in expression during Se deprivation. Unequal distribution of Se among selenoproteins means that limited Se availability can result in a loss of expression of some GPx family members. GPx3, along with GPx1, is ranked lowest in this hierarchy [47,48]. Hence their expression is very sensitive to decreasing Se levels and disappears rapidly in response to Se deficiency, due to nonsense-mediated mRNA decay (details of selenocysteine translational regulation are further reviewed in [22,49,50]).

In addition to Se-dependent translation, GPx3 is also regulated at the transcriptional level. GPx3 has been demonstrated to be under the regulation of peroxisome proliferator-activated receptor γ (PPARγ) [51,52]. As illustrated in Section 2.4, sustained loss of PPARγ in response to oxidative stress leads to GPx3 downregulation in several chronic diseases [51,52,53]. In addition, the *GPX3* promoter contains binding sites for hypoxia-inducible factor 1 (HIF-1) and the specificity protein 1 (Sp1) transcription factor, as well as a metal response element (MRE) and an antioxidant response element (ARE) [25]. Hypoxia was shown to be a strong inducer of *GPX3* promoter activity in the renal clear cell carcinoma cell line Caki-2 [25], and GPx3 expression is an important component of the antioxidant defense mechanisms that protect rats from acute hypobaric hypoxia exposure [54]. Interestingly, this is in opposition to the regulation of GPx1, transcription of which is inhibited in response to hypoxia [55]. The presence of MRE and AREs in the *GPX3* promoter suggest that GPx3 is a stress-responsive antioxidant enzyme. However, there are conflicting reports on the role oxidants play in the transcriptional regulation of GPx3. In lung epithelial cells, various ROS inducers were shown to stimulate GPx3 mRNA expression [56]. However, this ROS-mediated regulation could not be demonstrated in Caki-2 cells [25]. The Nrf2 stress response transcription factor, which binds to AREs, is an important regulator of tumor cell survival during metastatic progression and regulates a number of antioxidants important in this process [57], including GPx2 [58]. However, direct binding of Nrf2 to the *GPX3* promoter ARE at position −148 to −158 could not be experimentally established in response to treatment with the protease inhibitor MG132 in human umbilical vein endothelial cells (HUVECs) [59]. Interestingly, the molecular machinery required for selenocysteine translation has been demonstrated to be under the regulation of oxidative stress [60]. It is unclear if these stress response pathways contribute to the regulation of GPx3 in tumor cells with observed increases in GPx3 expression. In cancers that display low GPx3 expression, hypermethylation of the *GPX3* promoter has been demonstrated as a mechanism for GPx3 downregulation, which is further discussed in Section 3.2 [61,62,63].

### 2.3. GPx3 Localization

GPx3 is the major extracellular GPx isoform. The kidney is the predominant tissue that contributes to plasma GPx3 expression, which is evident by the fact that systemic GPx3 levels and activity are significantly decreased in patients following nephrectomies, and rescued after kidney transplantation [24,64,65,66,67]. In the kidney, GPx3 is primarily expressed in parietal cells of the Bowman’s capsule and the proximal tubular epithelium at the basolateral membrane [64,65]. Since the GPx3 protein was initially isolated from plasma it is also known as GPx-P, GSHPx-3 or extracellular eGPx [24,66,67]. It should be noted that early studies investigating glutathione peroxidase activity in patient blood also measured erythrocyte GPx activity, which is largely represented by GPx1 [24,38]. Other tissues with high GPx3 expression include the lung, heart, liver, breast, placenta, pancreas, thyroid, skeletal muscle, pancreas, brain, intestine, adipose and epididymis, and high levels can be found in associated extracellular fluids, such as milk and bronchioalveolar lavage fluid [32,47,65,68,69,70]. GPx3 in the thyroid is produced by the thyroid follicular cells and released into the colloid space, presumably to protect the thyrocytes from oxidative damage from excess H_2_O_2_ not utilized for iodination [71]. GPx3 expression is also high in the epididymis, which was shown to be under androgen control [72]. Interestingly, it was found that epididymal cells do not secrete GPx3, which is in contrast to the findings of secreted GPx3 protein in uterine flushings of female mice [72]. In addition to being secreted into extracellular fluids, GPx3 can also have localized roles and bind to the basement membranes of certain organs where it is expressed, including the kidney, lung, and intestine [35]. The binding sites for GPx3 appear to be cell specific. For example, in the lungs, GPx3 binds only to the basement membrane of type II pneumocytes but not type I pneumocytes [35]. GPx3 also appears to preferentially bind to the basement membranes of renal cortical epithelial cells [35,38]. However, more research is needed to determine the molecular mechanism of GPx3 binding and function of GPx3 at basement membranes of specific cell types. In summary, the majority of GPx3 circulating in plasma is produced by the kidneys, while other organs express GPx3 locally in a cell type and sex specific manner.

### 2.4. Disease Association with Aberrant GPx3

Several diseases have been either positively or negatively associated with GPx3 expression and activity. Section 3 will further discuss the role of GPx3 in cancer. In general, low plasma GPx3 expression strongly correlates with decreased systemic Se concentrations. Many diseases are associated with Se deficiency, although excess Se can also lead to toxicity. As stated before, GPx3 translation along with GPx1 is most vulnerable to Se deprivation. Therefore, plasma GPx activity is a good indicator of Se deficiency [65]. Se deficiency, and in turn decreased expression of GPx3, has been shown to be an important risk factor of Kashin–Beck disease (KBD) [73]. Patients with KBD have increased *GPX3* gene methylation, leading to decreased GPx3 expression and increased chondrocyte apoptosis due to elevated oxidative stress [73]. Low plasma GPx3 levels are associated with renal failure [74], which is not surprising, given that the kidney is the primary organ responsible for plasma GPx3 secretion. Association between decreased GPx3 expression and increased risk of childhood and young adulthood arterial ischemic strokes, as well as arterial thrombosis and coronary artery disease have been reported [41,75,76,77]. This is likely due to the negative effects on NO levels, as demonstrated in *GPX3* knock-out mice [41]. Interestingly, GPx3 activity progressively decreases with age, with significant reduction occurring in people over 70 years old. It has been suggested that this age-associated decline in GPx3 activity could be a contributing factor to cardiovascular disease risk [78].

GPx3 expression is decreased in the lungs of cigarette smoke-induced chronic obstructive pulmonary disease (COPD) patients [51]. Conversely, GPx3 levels in the epithelial lining fluid of cigarette smokers is higher than non-smokers, probably in response to the increased exogenous ROS [56]. The contradiction between the two is possibly due to the chronic adaptations associated with COPD. While acute exposure to cigarette smoke likely induces the oxidative stress-dependent upregulation of GPx3, a decrease in PPARγ in response to chronic oxidative stress was shown to decrease GPx3 expression in COPD [51]. Similarly, the expression of GPx3 in diabetes and obesity in response to oxidative stress may be dependent on disease progression and chronic adaptations related to PPARγ levels, with higher GPx3 and PPARγ expression observed during disease onset, while levels of both decrease in advanced disease states [52,53]. It has been shown that decreased plasma GPx3 levels in obese patients may also be the result of pro-oxidative conditions, such as increased TNFα and hypoxia, which could be abrogated by N-acetylcysteine and PPARγ agonist treatment [79]. In contrast, in other conditions of redox stress and inflammation, GPx3 expression has been shown to increase [51,56,80], and high GPx3 levels in extracellular fluids are associated with chronic inflammatory diseases such as asthma [56] and metabolic syndrome [81].

Although more research is needed to identify the role, context-dependent changes, and regulatory pathways of GPx3 in the above diseases, it can be appreciated that decreased GPx3 expression is often associated with Se deficiency, kidney malfunction, or decreased PPARγ-regulated transcription. There is conflicting evidence related to the role and expression of GPx3 in diseases that involve chronic oxidative stress and inflammation, which reflects the studies related to the regulation of GPx3 by stress response pathways summarized in Section 2.2. While many of the above studies suggest that the primary role of GPx3 is to mitigate oxidative stress, the potential role of GPx3 as a modulator of redox signaling should also be considered. At sub-lethal levels, oxidants contribute to cellular signaling through the oxidation of redox-sensitive amino acids, which can affect the function of proteins such as transcription factors, kinases and phosphatases [82,83]. An example is the oxidation of active-site cysteine residues of protein tyrosine phosphatases, which leads to inhibition of phosphatase activity and consequential activation of kinase signaling cascades [83]. It is still debated if oxidants such as H_2_O_2_ directly oxidize these signaling proteins, or if enzymes such as peroxiredoxins act as redox relays (reviewed in [84]). Nonetheless, antioxidant enzymes, including the GPx family members, are important regulators of these signaling cascades by manipulating levels of oxidants at cellular redox signaling hubs [28,85]. We highlight several studies in Section 3.2 where GPx3 has been implicated in modulating the activity of redox signaling pathways in cancer cells [32,86,87,88,89,90].

## 3. Alterations in GPx3 Expression and Activity in Cancer

**Figure 2:**
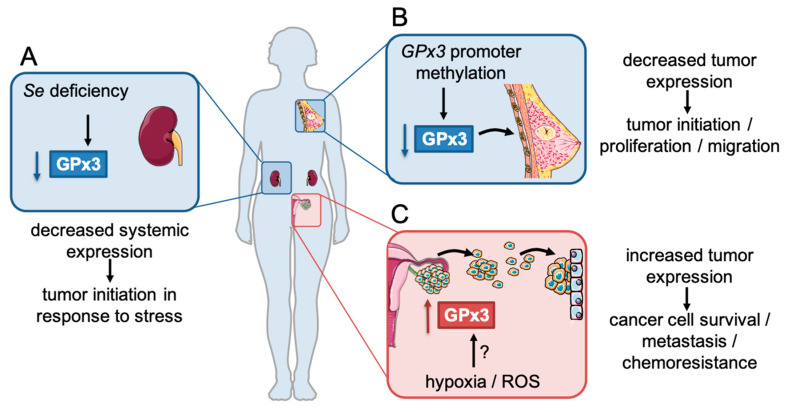
GPx3 has context-specific functions in cancer. (**A**) GPx3 expression can be altered at the systemic level in tumor patient plasma, likely as a consequence of Se deficiency. In animal models, decreased systemic GPx3 expression has been shown to be associated with tumor initiation in response to oxidative stress. (**B**,**C**) GPx3 expression is also altered within tumor tissues. (**B**) *GPX3* promoter hypermethylation has been associated with decreased GPx3 expression in some tumor tissues, including breast cancer. Decreased GPx3 expression has been associated with tumor initiation, proliferation, and migration, as a consequence of increased oxidative stress and pro-tumorigenic redox signaling. (**C**) The dichotomous role of GPx3 is highlighted by observations that GPx3 expression is also increased in some tumor tissues, including ovarian cancer. Increased GPx3 expression is associated with stemness, increased cancer cell survival, chemoresistance and metastatic progression. While the mechanisms of increased GPx3 expression remain to be fully elucidated, it is possible that stress response pathways, including hypoxia and oxidative stress, may contribute to GPx3 expression in these tumors.

Studies related to the role of GPx3 in cancer can be grouped into two categories. Section 3.1 summarizes studies examining changes in systemic plasma GPx3 levels, which are primarily associated with decreased expression by the kidneys in response to Se deficiency. Section 3.2 discusses the role of GPx3 expression changes within tumor tissues (Figure 2).

### 3.1. Changes in Systemic GPx3 Activity and the Role of Selenium Deficiency in Cancer

Many epidemiological studies have examined the role of Se deficiencies and concomitant changes in selenoproteins with disease outcome, and several studies have reported associations between Se deficiencies, decreased selenoprotein expression and cancer (reviewed in [91]). This has provided a strong rationale for clinical trials testing the effects of Se supplementation for chemoprevention [92]. While we will not focus extensively on this issue, a recent review of clinical trial data has revealed only limited benefits in chemoprevention with Se supplementation [92]. The lack of consideration of nutritional deficiencies of subjects at the start of some trials may also have offset results, as there is a suggestion that Se supplementation may only provide chemo-preventative benefits to subjects with low baseline Se levels. Moreover, there are indications that Se supplementation may also lead to deleterious effects. For example, there is a significant association between Se supplementation and increased risk of high-grade prostate cancer [92]. More in-depth and controlled studies are needed to elucidate the effectiveness of Se supplementation on cancer chemoprevention.

In cancer patients with metastatic disease, low Se levels and plasma GPx activity have been associated with enhanced lipid peroxidation, suggesting that loss of GPx3 contributes to systemic oxidative stress [93]. Decreased plasma GPx3 expression in cancer patients has been reported for several tumor types, including uterine and colorectal cancers [94,95]. Compared to healthy controls, GPx3 expression is also lower in both the plasma and tumor tissue of non-small-cell lung cancer (NSCLC), glioblastoma, and hepatocellular carcinoma (HCC) patients [88,96,97]. In HCC, low plasma GPx3 levels are associated with increased tumor recurrence and shorter disease-free survival periods after liver resection [88]. In the case of colon adenomas, patients have low basal serum Se and GPx3 levels that can be rectified with oral Se supplementation [98]. In papillary serous ovarian cancer systemic levels of GPx3 are decreased in a tumor-stage dependent manner, with late stage patients displaying a more pronounced decrease in plasma GPx3 levels [99]. However, as discussed in Section 3.2, high GPx3 expression within serous ovarian adenocarcinoma tumor tissues is conversely associated with poor prognosis and increased tumor stage [14]. Although oxidative stress from lipid peroxidation has been associated with increased risk of breast and esophageal cancer, changes in Se concentrations and GPx3 activity have not been linked to these cancers [100,101]. Instead, as illustrated in Section 3.2, changes in GPx3 expression may be associated with the tumor cells themselves. While low Se levels likely contribute to decreased plasma GPx3 expression by the kidney in cancer patients, the precise mechanisms contributing to Se depletion and plasma GPx3 loss are still unclear [93,95]. It is possible that Se and glutathione utilization by tumor tissues depletes systemic levels and influences plasma GPx3 protein translations and activity.

Studies using global *GPX3* knock-out mice have demonstrated that systemic loss of plasma GPx3 expression aids in tumor initiation. This is primarily observed when *GPX3-/-* mice are crossed with transgenic mice that have genetic alterations leading to tumor suppressor inhibition or oncogene activation, or when *GPX3-/-* mice are exposed to a chemical induced carcinogenesis model [102,103]. For example, when *GPX3-/-* mice are crossed with the transgenic adenocarcinoma of the mouse prostate (TRAMP) mice, loss of GPx3 increases the incidence of prostate cancer in this model [102]. Loss of GPx3 increases proliferation, decreases apoptosis and enhances Wnt/β-catenin signaling in tumor tissues [102]. Systemic GPx3 loss also enhances tumorigenesis in response to carcinogens. When *GPX3-/-* mice are exposed to azoxymethane/dextran sodium sulfate (AOM/DSS), loss of GPx3 accelerates the development of colonic high-grade dysplasia and increases expression of inflammatory markers in this colitis inflammatory carcinogenesis model [103]. No increases in ROS levels or DNA damage were observed in the *GPX3* knock-out mice prior to carcinogen treatment, suggesting that GPx3 loss primarily contributes to carcinogenesis in the context of exogenous stress [103]. The above studies demonstrate that low serum GPx3 levels are often observed in tumor patients and that this can contribute to tumor onset in the context of underlying driver mutations or carcinogenic stimuli. However, it is still unclear if systemic GPx3 loss is a major contributing risk factor for cancer development in response to other pro-tumorigenic stimuli, such as chronic inflammation and ionizing radiation. It is also not known if compensatory mechanisms are activated in response to GPx3 loss. For example, there is a suggestion that systemic Se deficiency can drive Nrf2-dependent gene expression, which could result in compensatory up-regulation of alternate antioxidant enzyme pathways [104].

In addition to Se deficiency, association studies of single nucleotide polymorphisms (SNP) in the *GPX3* gene suggest that certain *GPX3* SNPs may be associated with colorectal and gastric cancers. *GPX3* rs736775 C allele indicated better survival in colorectal cancer, and in gastric cancer, *GPX3* rs736775 TC/CC may be considered a prognostic marker since it was associated with improved overall survival in patients that received platin and fluorouracil-based adjuvant chemotherapy [105,106]. *GPX3* rs3805435 heterozygote G allele, rs3828599 heterozygote T allele, and rs2070593 heterozygote A allele are shown to be associated with decreased risk for gastric cancer as well [107]. In thyroid cancer, *GPX3* rs3805435 heterozygote G allele or rs3828599 heterozygote T allele may have a protective effect for differentiated thyroid cancer (DTC), specifically in an older population over 45 years of age. Conversely, the rs8177412 heterozygote C allele SNP is associated with increased risk for DTC [108]. These SNPs are primarily found in non-coding regions of the *GPX3* gene and their positive association with increased cancer risk is primarily thought to be due to their effects on GPx3 mRNA down-regulation [107].

### 3.2. Altered GPx3 Expression and Role in Tumor Tissues

**Figure 3:**
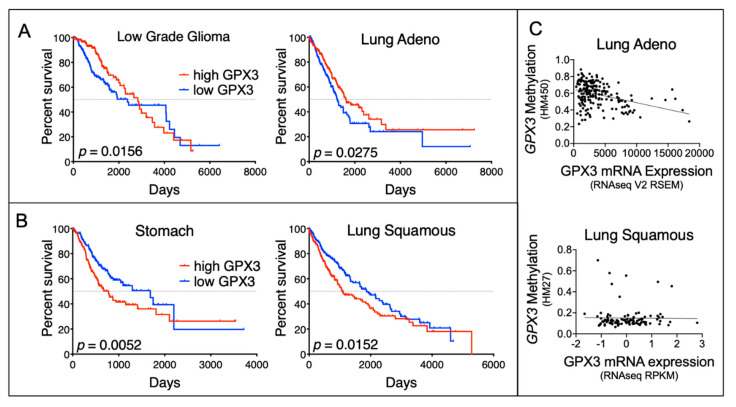
The dichotomous role of GPx3 in cancer is illustrated by the divergent associations between GPx3 expression and patient survival. (**A**) Low-grade glioma and lung adenocarcinomas are two examples where low GPx3 predicts worse overall patient survival. (**B**) Conversely, high GPx3 predicts worse overall patient survival in stomach and lung squamous cell carcinomas. (**C**) In cancers where low GPx3 expression is associated with poor outcome, such as lung adenocarcinoma, GPx3 mRNA expression (RNA-sequencing by expectation maximization [RSEM] values) correlates negatively with methylation of the *GPX3* gene (Illumina HumanMethylation450 Bead Chip; Pearson’s r = −0.3693, *p* < 0.0001). Conversely, in lung squamous cell carcinomas there is no association between GPx3 expression (expressed as Reads per Kilobase Transcript values from RNAseq) and *GPX3* methylation (Illumina Infinium Human Methylation 27 array). Patient overall survival data, mRNA GPx3 expression and *GPX3* methylation data were obtained from the Cancer Genome Atlas (TCGA) using cBioportal.org [120,121]. Kaplan–Meier curves were generated using GraphPad Prism software and curves statistically compared using the log rank test (Mantel–Cox).

As mentioned above, antioxidant enzymes often display dichotomous expression and have context-dependent roles within cancer cells [6,8,9,10], which are similarly observed for GPx3. Several studies have demonstrated that loss of GPx3 expression within tumor tissues is associated with poor patient prognosis and chemotherapeutic resistance (Table 1) [61,62,63,86,87,109,110,111,112,113,114,115]. Low tumor GPx3 expression can also predict patient outcomes. For example, data from the Cancer Genome Atlas (TCGA) demonstrate that low expression of GPx3 is associated with poor survival in lung adenocarcinoma and low-grade glioma (Figure 3A). However, in other tumor tissues GPx3 expression is elevated (Table 2) [14,116,117,118,119], and high expression is associated with poor patient outcomes in cancers such as stomach and lung squamous cell carcinomas (Figure 3B). Below we summarize the major findings from studies demonstrating both tumor suppressive and pro-tumorigenic properties of GPx3.

As summarized in Table 1, several studies have reported a decrease or loss of GPx3 expression in tumor tissues [61,62,63,86,87,109,110,111,112,113,114,115]. In many of these cancers, downregulation of GPx3 parallels cancer progression, and is often associated with poor patient outcome [62,90,109,110,123]. Loss of GPx3 expression has been linked to promoter hypermethylation in several tumor tissues [61]. *GPX3* gene methylation is usually biallelic, as seen in esophageal and endometrial cancers [62,63]. Examining available TCGA methylation data sets also demonstrates that GPx3 expression levels negatively correlate with increased *GPX3* gene methylation in tumor specimens such as lung adenocarcinoma (Figure 3C). Conversely, in lung squamous cell carcinomas, where high GPx3 expression is associated with poor patient survival (Figure 3B), there is no association between GPx3 expression and methylation (Figure 3C). In hepatocellular carcinoma, it was found that cells immediately adjacent to tumor tissues also display decreased GPx3 expression, with the authors suggesting that pre-cancerous lesions may already display GPx3 loss [88]. However, in clear cell renal cell carcinoma (ccRCC), *GPX3* methylation and GPx3 loss of expression were not observed in adjacent normal renal tissue [123]. In addition to gene methylation, *GPX3* gene deletions have been reported in prostate cancer, occurring in 39% of samples studied [109]. Screening TCGA data sets for somatic mutational signatures suggests that only a very low number of tumor specimens have missense mutations in the GPX3 coding region (somatic mutation rate of TCGA tumors: 0.3%; cBioPortal.org [120,121]). It has not been investigated if these have functional consequences, but due to their low frequency it is unlikely that somatic *GPX3* mutations contribute significantly to observed decreases in GPx3 expression and activity in tumor cells. Further, it is presently unclear if a loss of GPx3 drives compensatory increases in other antioxidant enzymes in tumor cells. However, in colorectal cancer cell culture studies, knock-down of GPx3 failed to induce compensatory increases in GPx1 or GPx2 [119].

Mechanistic studies have started to identify the tumor suppressive function of GPx3 in cancers where GPx3 is known to be downregulated (Table 1). GPx3 overexpression decreases clonogenic growth, *in vivo* xenograft tumor size, and metastasis of prostate cancer cells [109]. In cell culture studies, GPx3 overexpression similarly inhibits proliferation and invasion of lung cancer, HCC, and esophageal squamous cell carcinoma cells [86,88,90]. The anti-tumor properties of GPx3 have been largely associated with a loss of oxidant scavenging, concomitant increases in oxidative stress and pro-tumorigenic changes including genomic instability, as well as redox dependent signaling (summarized in Table 1) [109]. For example, it was shown that re-expression of GPx3 in lung cancer cell lines suppressed proliferation, migration and invasion by inhibiting the ROS-mediated activation of NF-kB signaling [86]. Re-expression of GPx3 inhibits the Erk-dependent activation of NF-κB, which leads to cyclin B1 inhibition and G2/M cell cycle arrest [86], as well as inhibition of epithelial-mesenchymal transition (EMT) by downregulating the Erk-NF-κB-SIP1 signaling axis [87,88]. GPx3 can also suppress the expression of matrix metalloproteinase 9 (MMP-9) by deactivating the focal adhesion kinase (FAK)-AKT pathway, leading to decreased invasiveness of esophageal squamous cell carcinoma cells [90]. The antitumor effects of GPx3 were also shown to be elicited by inhibiting HIF-1α and HIF-2α in melanoma cells as a consequence of increased ROS scavenging by GPx3 [89]. In prostate cancer, GPx3 overexpression induces downregulation of the oncogene *c-met*, although the mechanisms of this were not explored [109]. Many of the above studies demonstrate that the anti-tumor effects of GPx3 are dependent on its downregulation of oxidant-regulated pro-tumorigenic signaling pathways. An alternate tumor suppressive function for GPx3 was discovered in prostate cancer cell lines. Here, cytoplasmic-localized GPx3 was found to directly interact with p53-induced gene 3 (PIG3), which is activated by p53 and essential for p53-mediated apoptosis and response to DNA damage [131,132,133,134]. Induced expression of GPx3 conversely results in increased production of ROS and caspase-3 activity, as well as UV-induced apoptosis [128]. This study suggests a pro-oxidant role for the cytosolic form of GPx3, while the extracellular form lacking PIG3 interaction functions as an antioxidant enzyme [128].

Although a loss in GPx3 expression is associated with several tumor types (Table 1), reports also demonstrate that GPx3 expression is increased in some cancer cells and may aid in tumor progression (Table 2) [116,117,118,119]. For example, GPx3 is highly expressed in the clear cell adenocarcinoma subtypes, including renal and ovarian clear cell carcinomas [117,118]. The necessity of an extracellular antioxidant such as GPx3 in clear cell carcinomas, which are associated with highly secretory phenotypes, may be of significance and needs further study. Although some studies have demonstrated a decrease in GPx3 expression in ccRCC due to hypermethylation [109], it has also been shown that ccRCCs are highly reliant on the glutathione system for survival in response to nutrient and oxidative stress [123]. Using an RNAi screen, it was found that ccRCC cells were highly vulnerable to the depletion of both GPx3 and GPx4, loss of which increased susceptibility to lipid peroxidation and ferroptosis [117]. While the mechanism behind overexpression of GPx3 in patients with clear cell ovarian adenocarcinoma is unclear, GPx3 was shown to contribute to platinum and taxane-based chemoresistance [118,130]. Similarly, it was found that colorectal cancer cells with low GPx3 expression are also more sensitive to chemotherapies such as oxaliplatin and cisplatin, while cells with high GPx3 expression have increased resistance to platinum-based therapy [119]. This demonstrates that tumors with high GPx3 expression may have a survival advantage in response to exogenous stressors, including chemotherapeutics.

Knock-down of GPx3 in ovarian cancer cells inhibits anchorage-independent survival and enhances cell death in response to high dose ascorbate, a pro-oxidant at mM doses [14]. This suggests that GPx3 protects ovarian cancer cells during metastatic progression within malignant ascites, and in response to exogenous sources of ROS. Survival in anchorage independence is also associated with enrichment of stem cells, and it was shown that GPx3 expression significantly correlates with the frequency of leukemic stem cells in the *Hoxa9*+*Meis1*-induced leukemia model [116]. It has been found that enhanced expression of GPx3 in leukemic stem cells is associated with *GPX3* promoter hypomethylation, suggesting that the epigenetic regulation of GPx3 may be reversible and context dependent. Moreover, the investigators demonstrated that GPx3 is necessary for self-renewal capacity and optimal repopulation of cells, and is associated with poor patient outcome [116]. A pro-tumorigenic function has similarly been demonstrated for the gastrointestinal GPx isoform, GPx2 [11], and the above studies suggest that there could also be a need for enhanced scavenging of hydro- and soluble lipid hydroperoxides in the extracellular tumor environment by GPx3. This may be an important survival adaptation, specifically to enable tumor progression and metastasis in hostile tumor microenvironments. Moreover, it suggests that increased extracellular GPx3 activity confers survival advantages to tumor cells when exposed to exogenous insults, such as chemotherapeutic agents.

## 4. Conclusions and Perspectives

In conclusion, similar to other antioxidants, GPx3 has a dichotomous role in cancer as both a tumor suppressor and a pro-survival protein during tumor progression (Figure 2; Table 1 and Table 2). Further studies are required to understand the complex roles of GPx3 in cancer, especially its dichotomous regulation and role in different cancer types. As such, the function of GPx3 as an extracellular or basement membrane localized antioxidant enzyme has not been explored in detail. The consequences of either high or low GPx3 expression in tumors may be related to the tumors’ need to scavenge oxidants in the extracellular environment. One might hypothesize that tumors with high GPx3 expression are specifically in need of extracellular hydroperoxide or soluble lipid hydroperoxide scavenging in tumor-associated fluids. One such fluid includes the abdominal accumulation of ascites, which is strongly associated with abdominal-localized tumors such as stomach, liver, and ovarian cancers [135,136]. It is intriguing to note that many of the tumor types that demonstrate poor patient survival related to high tumor GPx3 expression, including stomach (Figure 3B) and ovarian cancers [14], are also known to produce malignant ascites [135,136]. One possible explanation for the need of GPx3 in tumor-derived ascites is the observed increase in lipid peroxides in this fluid. For example, in ovarian cancer ascites, soluble lipid peroxides were increased in patients that were refractory to platinum-based chemotherapy [137]. The identification and role of specific substrates of GPx3 in tumors, in particular soluble lipid hydroperoxides in the tumor microenvironment and basement membranes, require further attention. While the studies summarized above demonstrate that GPx3 can influence intracellular redox signaling, it still remains largely unclear if enhanced or reduced oxidant scavenging by GPx3 has consequences on the extracellular tumor environment and tumor associated cells. For example, SOD3 has been shown to elicit its anti-invasive function partly by inhibiting the oxidation-induced fragmentation of extracellular matrix proteins and preventing oxidation of the MMP inhibitor membrane-type MMP (MT-MMP) [6]. It remains unknown if GPx3 similarly inhibits the oxidation of matrix components.

Another issue to consider is how the extracellular tumor environment contributes to the regulation of GPx3 activity. As such, the availability of GSH may also be an important regulator of GPx3 activity. Interestingly, multidrug resistance proteins, which are often upregulated in cancer, also transport GSH [138,139]. This suggests that an enhanced efflux of GSH into the extracellular space could contribute to increased GPx3 activity in the tumor microenvironment. As such, one might also speculate that amino acid precursors of GSH synthesis, and compounds such as N-acetylcysteine (NAC) could amplify the activity of GPx3 [140]. Given that NAC has been shown to promote metastasis of some tumor types [8,141], it is of interest to determine if the presence of GPx3 in the tumor microenvironment is necessary for the pro-metastatic actions of NAC.

Although further studies are needed to better understand the role of GPx3 in cancer at both the systemic and tumor cell levels, targeting of GPx3 may be an attractive therapeutic strategy. This has been primarily addressed in tumors displaying low GPx3 expression. Several pre-clinical studies have demonstrated that re-expressing GPx3 in cancer cells is beneficial to inhibit proliferation and metastatic behavior [86,88,90]. Moreover, delivery of GPx3 to hepatocellular carcinoma tumors using iPSC-derived mesenchymal stem cells reduces tumor growth in cell culture and xenograft mouse model in vivo [88]. Although not a targeted approach, DNA demethylation agents are also able to increase expression and rescue the tumor suppressive functions of GPx3 [62,63]. While demethylating agents, such as azacitidine, have produced mixed results in the clinic, they are used for treatment of myeloid leukemia, and next generation agents and combination therapy regimes are currently in clinical trials [142]. Given that *GPX3* hypermethylation is a phenotype of a number of tumors, it is of interest to further assess if reversal of GPx3 expression contributes to the anti-tumor effects of these agents. Alternative approaches might include the use of GPx mimetics, such as ebselene, which have shown to rescue effects of GPx3 knock-down on chronic kidney disease [27,41]. However, given that many tumor cells rely on other GPx isoforms and glutathione for pro-survival ROS scavenging during tumor progression [11,12], a more targeted approach to specifically mimic extracellular GPx3 activity may need to be developed. An alternative to the above strategies is to utilize the lack of GPx3 expression in tumors as a therapeutic advantage. Screening for GPx3 low tumors might predict a patient’s chemotherapy response, as loss of GPx3 expression has been shown to confer sensitivity to platinum- and taxane-based chemotherapeutics [118,119,130]. Moreover, cells that lack GPx3 expression are more susceptible to cytotoxicity by pro-oxidants, such as high dose ascorbate [14]. While further studies are necessary, GPx3 tumor expression could be an important biomarker to determine the efficacy of pro-oxidant therapies in the future.

## Figures and Tables

**Table 1:** Examples of studies demonstrating GPx3 downregulation in tumor tissues. Mechanisms of GPx3 expression loss and implications of GPx3 downregulation on tumor cells and pro-tumorigenic signaling are summarized if reported (N/D: not determined).

Cancer	Causes of Downregulation	Implications of Downregulation	Tumor Suppression Mechanisms
Bladder	Gene hypermethylation	Cancer progression, potential biomarker [61,122]	N/D
Breast	Gene hypermethylation	Cancer progression [114]	N/D
Cervical	Gene hypermethylation	Cancer progression and metastasis, poor patient outcome [110]	N/D
Clear cell renal cell carcinoma	Gene hypermethylation	Cancer progression, potential biomarker [123]	N/D
Colorectal	Gene hypermethylation	Increased chemosensitivity [119]	N/D
Endometrial	Gene hypermethylationGene deletion [63]	N/D	N/D
Esophageal	Gene hypermethylation	Cancer progression, poor patient outcome, potential biomarker [62,90]	Suppress expression of matrix metalloproteinase 9 by deactivating the FAK/AKT pathway [90]
Gastric	Gene hypermethylation	Cancer progression [87]	Inhibit epithelial-mesenchymal transition (EMT) and regulating the NF-κB/Wnt/JNK pathway [87]
Head and Neck	Gene hypermethylation	Chemoresistance, poor patient outcome [61]	N/D
Hepatocellular	Gene hypermethylation	Cancer progression, poor patient outcome [88,124]	Inhibit EMT through the Erk-NF-κB-SIP1 pathway [88]
Lung	Gene hypermethylation	Poor patient outcome, potential biomarker [125]	Inhibit activation of Erk-NF-κB-cyclin B1 pathway [86]
Melanoma	Gene hypermethylation	Cancer progression, poor patient outcome [111]	Inhibit expression of hypoxia-inducible factors 1α and 2α [89]
Multiple myeloma	Gene hypermethylation	Poor patient outcome [126]	N/D
Myeloid leukemia	Gene hypermethylation	Favorable/intermediate karyotype [112,127]	N/D
Prostate	Gene hypermethylationGene deletion	Cancer progression, poor patient outcome [109]	Downregulation of *c-met* [109] Interaction with p53-induced gene 3 to induce apoptosis [128]
Thyroid	Gene hypermethylation	Cancer progression and metastasis [113]	N/D

**Table 2:** Examples of studies demonstrating increased GPx3 expression in tumor tissues, and implications of GPx3 upregulation.

Cancer	Causes of Upregulation	Implications of Upregulation
Clear cell renal cell carcinoma	N/D	Cancer maintenance [117]
Colorectal	N/D	Chemoresistance [119]
Leukemia stem cells	Gene hypomethylation	Poor patient outcome [116]
Myeloid leukemia	N/D	Cancer progression, poor patient outcome [116,129]
Ovarian cancer	N/D	Cancer cell survival under anchorage independence and oxidative stress [14]
Ovarian clear cell adenocarcinoma	N/D	Chemoresistance [130]
